# Complete Genome Sequence of Clostridioides difficile Ribotype 255 Strain Mta-79, Assembled Using Oxford Nanopore and Illumina Sequencing

**DOI:** 10.1128/MRA.00935-19

**Published:** 2019-10-17

**Authors:** Jennifer K. Spinler, Anne J. Gonzales-Luna, Sabeen Raza, Jessica K. Runge, Ruth Ann Luna, Tor C. Savidge, Kevin W. Garey

**Affiliations:** a Department of Pathology & Immunology, Baylor College of Medicine, Houston, Texas, USA; b Texas Children’s Microbiome Center, Department of Pathology, Texas Children’s Hospital, Houston, Texas, USA; c University of Houston College of Pharmacy, Houston, Texas, USA; Loyola University Chicago

## Abstract

Hybrid *de novo* assembly of Illumina/Nanopore sequence data produced a complete circular sequence of the chromosome for a Clostridioides difficile ribotype 255 (RT255) isolate from an elderly patient with recurrent C. difficile infection (CDI). This provides a high-quality representative sequence for the RT255 lineage.

## ANNOUNCEMENT

Clostridioides difficile is the leading cause of antibiotic-associated diarrhea and poses an urgent public health threat with >300,000 nosocomial infections in the United States each year (1). Recent epidemiological surveys indicate that C. difficile ribotype 255 (RT255) has emerged as the fifth most common RT in Texas and supersedes hypervirulent RTs 027 and 078 in Houston, TX (2). We report the complete genome sequence of an RT255 strain, C. difficile Mta-79, to serve as a high-quality reference sequence for the RT255 lineage.

C. difficile Mta-79 was obtained from an 80-year-old female during her third recurrent episode of community-onset health care facility-associated C. difficile infection (CDI) in Houston, TX. Cryo-frozen isolates were incubated on cycloserine-cefoxitin fructose agar (CCFA) plates under anaerobic conditions for 48 h. Isolates were confirmed to be C. difficile by Gram staining, typical odor, and presence of C. difficile antigen on microscreen latex agglutination (Microgen Bioproducts). Genomic DNA was extracted using the MasterPure complete DNA and RNA purification kit (catalog number MC85200; Lucigen). The same DNA prep was used for both Illumina and Nanopore sequencing. For Illumina sequencing, libraries of fragmented genomic DNA were prepared using a NEXTflex rapid DNA-seq kit (catalog number NOVA-5149-02; Bioo Scientific). Paired-end reads (1,117,792 2 × 150-bp reads) were generated on the MiSeq platform (Illumina, San Diego, CA, USA) using the Illumina MiSeq reagent kit v2 (catalog number MS-102-2002) and the PhiX control kit v3 (catalog number FC-110-3001). Raw reads were quality filtered and adapter trimmed with Trimmomatic v0.36 (3). Oxford Nanopore Technologies (ONT) sequencing was carried out on libraries prepared with ONT’s rapid barcoding kit (catalog number SQK-RBK004) using a MinION device with flow cell type R9.4.1 (catalog number FLO-MIN106D). Guppy v3.1.5 was used to base call, quality filter (minimum Q score, 10), demultiplex, barcode, and quality trim the 58,759 ONT reads. Multilocus sequence typing analysis of Illumina reads with SRST2 (4) classified C. difficile Mta-79 as sequence type 34 (ST34). Nanopore and Illumina reads were combined for hybrid *de novo* assembly using Unicycler v0.4.8-beta (5) in the conservative assembly mode. One circular chromosome of 4,123,685 bp (28.68% G+C content) resulted. The average genome coverage for short- and long-read sequences was 77.3× and 39.5×, respectively, as calculated using BBMap v38.68 (https://sourceforge.net/projects/bbmap/). The assembly was annotated with the NCBI Prokaryotic Annotation Pipeline (6) and resulted in 3,634 protein-coding genes, 32 rRNA operons, and 9 CRISPR arrays.

### Data availability.

The genome sequence for C. difficile Mta-79 (RT255) has been deposited in NCBI under BioProject PRJNA556848. The Illumina paired-end FASTQ and ONT base-called FASTQ files are available in the Sequence Read Archive under accession numbers SRR9850531 and SRR9850530, respectively. The annotated genome is listed under the NCBI accession number CP042267.
